# An Unusual Presentation of Endometriosis as an Ileocolic Intussusception with Cecal Mass: A Case Report

**Published:** 2018

**Authors:** Neda Nozari, Maryam Shafiei, Soheila Sarmadi

**Affiliations:** 1- Gastrointestinal Ward, Yas Hospital, Tehran University of Medical Sciences, Tehran, Iran; 2- Department of Surgery, Yas Hospital, Tehran University of Medical Sciences, Tehran, Iran; 3- Department of Pathology, Yas Hospital, Tehran University of Medical Sciences, Tehran, Iran

**Keywords:** Bowel, Endometrioma, Endometriosis, Intestinal obstruction

## Abstract

**Background:**

Bowel endometriosis affects about 3.8–37% of women with endometriosis diagnosis. Most of the time endometriosis involves the recto-sigmoid. Right colon involvement is not common in endometriosis and also a few studies have reported obstructive endometriosis of bowel. Here, a case of endometriosis was reported with the ileocolic intussusception and cecal mass.

**Case Presentation:**

A 32y old woman was referred to Yas hospital due to severe low abdominal pain and vomiting. Ultrasonographic examination of her pelvis revealed bilateral ovarian cysts. Abdominal erect X-ray showed dilatation of small bowel segments. Diagnostic colonoscopy showed one small ulcer with the pressure effect of mass like lesion at cecum. The patient was taken to the operating room for excision of the mass; as a result the ileocolic intussusception was seen. After reduction, a firm mass was recognized at cecum so the ileocecal resection was performed. In pathologic examination of mass, endometriosis was reported. The postoperative period was uneventful.

**Conclusion:**

The diagnosis of bowel endometriosis is sometimes difficult. The case of bowel obstructive endometriosis is rare. Surgical excision of bowel endometriosis is necessary for symptomatic patients with bowel obstruction. Bowel endometriotic nodules are excised by nodulectomy or segmental resection.

## Introduction

It has been estimated that 8–15% of young ladies have endometriosis (1–3). Bowel endometriosis affects about 3.8–37% of women with endometriosis diagnosis (1, 4, 5). Most of the time, endometriosis involves the recto-sigmoid (80% of cases) and very rarely the small intestine (5, 6). Bowel endometriosis can mimic other bowel diseases including inflammatory bowel disease, ischemic colitis, or even malignant tumors. Bowel endometriosis sometimes leads to surgical resections (1, 5, 6). The gastrointestinal symptoms are non-specific such as abdominal pain, nausea and vomiting (5–7). Right colon involvement is not common in endometriosis and also few studies have reported obstructive endometriosis of bowel. Here, a case of endometriosis was reported with the ileocolic intussusception and cecal mass.

## Case Presentation

**Figure 1.**
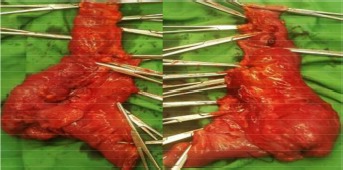
Postoperative specimen of the bowel. The mass is visible at cecum segment

A 32y old woman was referred to Yas hospital due to severe low abdominal pain and vomiting on May 2017. Ultrasonographic examination of her pelvis revealed bilateral ovarian cysts. During the 5 days before the admission, she had experienced severe right lower abdominal pain and vomiting especially after a meal. She had a long history of dysmenorrhea and, one cesarean delivery 3 years before. She was taking no medication. Her physical examination report included the temperature of 37°*C*, systolic blood pressure of 100 *mmHg* and heart rate of 120 beats per minute. The right lower quadrant of her abdomen was tender along with hypoactive bowel sounds. Laboratory data reported leukocytosis (16×10^9^/L) with neutrophilia, C-reactive protein of 20 *mg/dl* and erythrocyte sedimentation rate of 60 *mm/hr*. Abdominal erect X-ray showed dilatation of small bowel segments. Colonoscopy was requested by gastroenterologist for finding the cause and excluding colon neoplasm. Diagnostic colonoscopy showed one small ulcer (8 *mm*) with the pressure effect of mass like lesion at cecum and scope couldn’t find the ileocecal valve. It was thought that a mass like lesion had caused the ileum obstruction. Colonoscopic biopsies were taken and histopathological examination revealed endometriosis. Afterwards, the patient was taken to the operating room for excision of the mass. At the exploration of the abdominal cavity, adequate exposure was attained by a vertical incision across the midline of the abdomen with a transverse extension to the right. The ileocecal part was covered by the omentum and was adherent to the abdominal wall. The ileocolic intussusception was seen without ischemic changes. Reduction of the intussusception was performed at first. After reduction, a firm mass was recognized at cecum, located close to the ileocecal valve. Then, the ileocecal resection was performed (Figure 1). Pathological examination confirmed endometriosis.

The postoperative period was uneventful and she was discharged on the third postoperative day. She was doing well at the 6 months follow up.

### Consent:

Written informed consent was obtained from the patient for publication of this case report and accompanying images.

## Discussion

Some studies have indicated misdiagnosis of endometriosis as irritable bowel syndrome in young ladies due to nonspecific symptoms of both (6, 8). The types of endometriosis include superficial peritoneal lesions, ovarian endometriosis, and deep infiltrating endometriosis (DIE) (9, 10). Most of the patients with ovarian endometriosis expect to find disease elsewhere and should be evaluated especially for bowel endometriosis (7, 11). Transvaginal ultrasound, magnetic resonance imaging or transrectal ultrasound can find lesions of endometriosis (4, 9). The standard diagnostic test for bowel endometriosis is the laparoscopic visualization of bowel lesions (5, 9, 10). The endometriotic nodules are common in rectosigmoid (80%) then ileum, appendix (0.8%) and cecum (3–5). Although some studies have indicated diagnosis of bowel endometriosis by colonoscopy, but colonoscopy (7% sensitivity and 98% specificity) is not a useful diagnostic procedure in evaluating patients with bowel endometriosis in most gynecology centers (1, 10). Because the incidence of colonoscopic finding is low even in DIE (4%) (10). Distortion, narrowing or inward bulging of the colon lumen, polyp or mass, and mucosal invasion such as erythema and nodularity are bowel endometriosis findings by colonoscopy (5, 10). Eccentric wall thickening is the most common picture (82%) of bowel endometriosis in colonoscopy (1, 10). Colonoscopic biopsies are small tissue for pathologist to report definite diagnosis. The diagnostic accuracy of colonoscopic biopsy is high when the mucosal invasion of endometriosis is seen macroscopically (1). The treatment options for bowel endometriosis depend on size and location of the lesion, stenosis of bowel lumen, severity of the symptoms, desire of patient to conceive and tolerability of hormonal therapies (4, 7). Hormonal therapies (Progestin, gonadotropin releasing hormone analogues and aromatase inhibitors) improve gastrointestinal symptoms in patients with bowel stenosis less than 60% if they do not wish to conceive (4, 5). But hormonal therapies are not effective for bowel endometriosis and do not prevent disease progression. Careful monitoring should be considered for patients receiving long-term treatment (4). Surgical excision of bowel endometriosis is necessary for symptomatic patients with bowel stenosis more than 60% (4, 5). Bowel endometriotic nodules are excised by nodulectomy or segmental resection (4, 12). Surgery is crucial for bowel obstructive endometriosis. Some studies have reported that the pregnancy rate is 49% after the surgery (5). Endometriosis has been reported in 15–44% of all laparotomies or laparoscopies in young ladies (2). Unfortunately, some studies have indicated unnecessary aggressive surgical resections in some patients with bowel endometriosis due to misdiagnosis as a neoplastic lesion (1, 6).

## Conclusion

The clinician should be aware that endometriosis can be multifocal and with an unusual presentation. The diagnosis of bowel endometriosis is sometimes difficult. Bowel obstructive endometriosis is rare and surgery is the main treatment. Bowel endometriotic lesions may be excised by segmental resection or nodulectomy.
